# Supplementary material for: The Nature and Extent of Plasmid Variation in Chlamydia trachomatis
Source: Microorganisms. 2020 Mar 6;8(3):373. doi: 10.3390/microorganisms8030373 (PMC7143637; doi:10.3390/microorganisms8030373)
Supplement: Supplementary file 1 [file microorganisms-08-00373-s001.zip › Jones et al Supplementary Data Files/Table S1.docx]

Supplementary data table 1: Metadata on *C. trachomatis* strains used in this investigation

| Strain | Clade | Genotype | Country in which Strain Isolated | Continent in which strain found | Body site strain isolated from | Sex of Person strain was sampled from | Year Strain Isolated | Accession Number (Genbank) | Accession number (ENA database) |
| --- | --- | --- | --- | --- | --- | --- | --- | --- | --- |
|  |  |  |  |  |  |  |  |  |  |
|  |  |  |  |  |  |  |  |  |  |
| A_5291 | ocular | A | Tanzania | 3.Africa | ocular | F | 2000 | HE603210 | ERS017901 |
| A_7249 | ocular | A | Tanzania | 3.Africa | ocular | M | 2000 | HE603211 | ERS017902 |
| A_D213 | ocular | A | Gambia | 3.Africa | ocular | M | 2001 |  | ERS177838 |
| A_MH10549 | ocular | A | Tanzania | 3.Africa | ocular | M | 2000 |  | ERS177738 |
| A_MH10648 | ocular | A | Tanzania | 3.Africa | ocular | F | 2000 |  | ERS177739 |
| A_MH10901 | ocular | A | Tanzania | 3.Africa | ocular | M | 2000 |  | ERS177740 |
| A_MH11715 | ocular | A | Tanzania | 3.Africa | ocular | M | 2000 |  | ERS177741 |
| A_MH11979 | ocular | A | Tanzania | 3.Africa | ocular | M | 2000 |  | ERS177742 |
| A_MH12023 | ocular | A | Tanzania | 3.Africa | ocular | F | 2000 |  | ERS177721 |
| A_MH1364 | ocular | A | Tanzania | 3.Africa | ocular | F | 2000 |  | ERS177731 |
| A_MH13849 | ocular | A | Tanzania | 3.Africa | ocular | M | 2000 |  | ERS177722 |
| A_MH14553 | ocular | A | Tanzania | 3.Africa | ocular | F | 2000 |  | ERS177723 |
| A_MH15048 | ocular | A | Tanzania | 3.Africa | ocular | F | 2000 |  | ERS177743 |
| A_MH15741 | ocular | A | Tanzania | 3.Africa | ocular | F | 2000 |  | ERS177744 |
| A_MH16005 | ocular | A | Tanzania | 3.Africa | ocular | M | 2000 |  | ERS177724 |
| A_MH16170 | ocular | A | Tanzania | 3.Africa | ocular | M | 2000 |  | ERS177745 |
| A_MH16665 | ocular | A | Tanzania | 3.Africa | ocular | M | 2000 |  | ERS177746 |
| A_MH17127 | ocular | A | Tanzania | 3.Africa | ocular | M | 2000 |  | ERS177747 |
| A_MH18843 | ocular | A | Tanzania | 3.Africa | ocular | F | 2000 |  | ERS177725 |
| A_MH18876 | ocular | A | Tanzania | 3.Africa | ocular | F | 2000 |  | ERS177748 |
| A_MH19657 | ocular | A | Tanzania | 3.Africa | ocular | M | 2000 |  | ERS177726 |
| A_MH19679 | ocular | A | Tanzania | 3.Africa | ocular | F | 2000 |  | ERS177749 |
| A_MH20130 | ocular | A | Tanzania | 3.Africa | ocular | F | 2000 |  | ERS177750 |
| A_MH20933 | ocular | A | Tanzania | 3.Africa | ocular | F | 2000 |  | ERS177751 |
| A_MH21571 | ocular | A | Tanzania | 3.Africa | ocular | F | 2000 |  | ERS177752 |
| A_MH23527 | ocular | A | Tanzania | 3.Africa | ocular |  |  |  | ERS177727 |
| A_MH24519 | ocular | A | Tanzania | 3.Africa | ocular | M | 2000 |  | ERS177753 |
| A_MH24640 | ocular | A | Tanzania | 3.Africa | ocular | F | 2000 |  | ERS177754 |
| A_MH24673 | ocular | A | Tanzania | 3.Africa | ocular | M | 2000 |  | ERS177728 |
| A_MH2497 | ocular | A | Tanzania | 3.Africa | ocular | F | 2000 |  | ERS177718 |
| A_MH25256 | ocular | A | Tanzania | 3.Africa | ocular | M | 2000 |  | ERS177729 |
| A_MH26862 | ocular | A | Tanzania | 3.Africa | ocular | F | 2000 |  | ERS177756 |
| A_MH27137 | ocular | A | Tanzania | 3.Africa | ocular | F | 2000 |  | ERS177757 |
| A_MH3234 | ocular | A | Tanzania | 3.Africa | ocular | F | 2000 |  | ERS177732 |
| A_MH34496 | ocular | A | Tanzania | 3.Africa | ocular | M | 2000 |  | ERS177758 |
| A_MH35739 | ocular | A | Tanzania | 3.Africa | ocular | M | 2000 |  | ERS177759 |
| A_MH4510 | ocular | A | Tanzania | 3.Africa | ocular | F | 2000 |  | ERS177733 |
| A_MH47300 | ocular | A | Tanzania | 3.Africa | ocular | F | 2000 |  | ERS177730 |
| A_MH53658 | ocular | A | Tanzania | 3.Africa | ocular | F | 2000 |  | ERS177760 |
| A_MH5368 | ocular | A | Tanzania | 3.Africa | ocular | F | 2000 |  | ERS177719 |
| A_MH5786 | ocular | A | Tanzania | 3.Africa | ocular | M | 2000 |  | ERS177720 |
| A_MH6446 | ocular | A | Tanzania | 3.Africa | ocular | M | 2000 |  | ERS177734 |
| A_MH7205 | ocular | A | Tanzania | 3.Africa | ocular | M | 2000 |  | ERS177735 |
| A_MH858 | ocular | A | Tanzania | 3.Africa | ocular | M | 2000 |  | ERS177716 |
| A_MH8910 | ocular | A | Tanzania | 3.Africa | ocular | M | 2000 |  | ERS177736 |
| A_MH9922 | ocular | A | Tanzania | 3.Africa | ocular | F | 2000 |  | ERS177737 |
| A_SA1 | ocular | A | Saudi_Arabia | 2.M.East_Asia | ocular | F | 1957 |  | ERS177777 |
| B_Aus25 | T1 | B-Ba | Australia | 1.Aus | ocular |  |  |  | ERS351392 |
| B_Aus28 | T1 | B-Ba | Australia | 1.Aus | ocular |  |  |  | ERS351377 |
| B_Aus3 | T2 | B-Ba | Australia | 1.Aus | unknown | unk |  |  | ERS153020 |
| B_Aus36 | T2 | B-Ba | Australia | 1.Aus | ocular |  |  |  | ERS351385 |
| B_Aus4 | T2 | B-Ba | Australia | 1.Aus | unknown | unk |  |  | ERS153021 |
| B_Aus5 | T2 | B-Ba | Australia | 1.Aus | unknown |  |  |  | ERS153022 |
| B_Aus6 | T1 | B-Ba | Australia | 1.Aus | unknown |  |  |  | ERS153043 |
| B_Fin101 | T2 | B-Ba | Finland | 4.Scand_Russia | endocervical | F | 2009 |  | ERS200114 |
| B_Fin203 | T2 | B-Ba | Finland | 4.Scand_Russia | endocervical | F | 2011 |  | ERS200083 |
| B_HAR36 | ocular | B-Ba |  |  |  |  |  |  | ERS153013 |
| B_Jali16 | ocular | B-Ba | Gambia | 3.Africa | ocular |  |  |  | ERS153015 |
| B_M48 | ocular | B-Ba | Gambia | 3.Africa | ocular | M | 2007 |  | ERS177817 |
| B_NL2 | T2 | B-Ba | Netherlands | 5.Europe | endocervical | F | 2001 |  | ERS161066 |
| B_Sou42 | T2 | B-Ba | UK | 6.UK | unk |  | 1985 |  | ERS161047 |
| Ba_Apache2 | ocular | B-Ba | USA | 7.N.America | ocular |  |  |  | ERS095032 |
| C_Aus10 | T1 | C | Australia | 1.Aus | unknown |  |  |  | ERS153023 |
| C_Aus30 | T1 | C | Australia | 1.Aus | ocular |  |  |  | ERS351383 |
| C_Aus33 | T1 | C | Australia | 1.Aus | ocular |  |  |  | ERS351384 |
| C_Aus8 | T1 | C | Australia | 1.Aus | unknown | unk |  |  | ERS161042 |
| C_Aus9 | T1 | C | Australia | 1.Aus | unknown | unk |  |  | ERS153045 |
| C_TW3 | ocular | C | Taiwan | 2.M.East_Asia | ocular |  | 1959 |  | ERS177778 |
| C_UW10 | ocular | C | Canada | 7.N.America | ocular | M? | 1964 |  | ERS177816 |
| D_9805238 | T2 | D | Indonesia | 2.M.East_Asia | cervical | F | 1998 |  | ERS373088 |
| D_Aus11 | T2 | D | Australia | 1.Aus | unk | unk |  |  | ERS153024 |
| D_Aus12 | T2 | D | Australia | 1.Aus | unk |  |  |  | ERS153044 |
| D_C32 | T2 | D | UK | 6.UK | Ur/Cx | F | 1986 |  | ERS177807 |
| D_Fin163 | T1 | D | Finland | 4.Scand_Russia | endocervical | F | 2010 |  | ERS200072 |
| D_Fin178 | T2 | D | Finland | 4.Scand_Russia | endocervical | F | 2010 |  | ERS200076 |
| D_Fin187 | T1 | D | Finland | 4.Scand_Russia | endocervical | F | 2010 |  | ERS200079 |
| D_HonLC4 | T1 | D |  | 8.S.America |  |  |  |  | ERS151260 |
| D_HPA314 | T1 | D | UK | 6.UK | cervical | F |  |  | ERS082979 |
| D_NL10 | T1 | D | Netherlands | 5.Europe | endocervical | F | 2001 |  | ERS133263 |
| D_NL11 | T2 | D | Netherlands | 5.Europe | endocervical | F | 2001 |  | ERS133264 |
| D_NL12 | T2 | D | Netherlands | 5.Europe | endocervical | F | 2001 |  | ERS133265 |
| D_NL13 | T2 | D | Netherlands | 5.Europe | endocervical | F | 2001 |  | ERS133266 |
| D_NL14 | T2 | D | Netherlands | 5.Europe | endocervical | F | 2001 |  | ERS133267 |
| D_NL15 | T2 | D | Netherlands | 5.Europe | endocervical | F | 2001 |  | ERS133268 |
| D_NL16 | T2 | D | Netherlands | 5.Europe | endocervical | F | 2001 |  | ERS161020 |
| D_NL17 | T2 | D | Netherlands | 5.Europe | endocervical | F | 2001 |  | ERS133269 |
| D_NL19 | T1 | D | Netherlands | 5.Europe | endocervical | F | 2001 |  | ERS133271 |
| D_NL32 | T2 | D | Netherlands | 5.Europe | endocervical | F | 2001 |  | ERS153040 |
| D_NL4 | T1 | D | Netherlands | 5.Europe | endocervical | F | 2001 |  | ERS161067 |
| D_NL5 | T1 | D | Netherlands | 5.Europe | endocervical | F | 2001 |  | ERS161068 |
| D_NL59 | T2 | D | Netherlands | 5.Europe | endocervical | F | 2001 |  | ERS153042 |
| D_NL6 | T1 | D | Netherlands | 5.Europe | endocervical | F | 2001 |  | ERS133259 |
| D_NL71 | T2 | D | Netherlands | 5.Europe | endocervical | F | 2001 |  | ERS161038 |
| D_NL8 | T1 | D | Netherlands | 5.Europe | endocervical | F | 2001 |  | ERS133261 |
| D_S1736 | T2 | D | Sweden | 4.Scand_Russia | endocervical | F | 2010 |  | ERS082968 |
| D_S1879 | T1 | D | Sweden | 4.Scand_Russia | urethra | M | 2010 |  | ERS082969 |
| D_S2130 | T2 | D | Sweden | 4.Scand_Russia | endocervical | F | 2010 |  | ERS075209 |
| D_S276I | T1 | D | Sweden | 4.Scand_Russia | endocervical | F | 2011 |  | ERS095024 |
| D_S3257 | T2 | D | Sweden | 4.Scand_Russia | endocervical | F | 2010 |  | ERS075213 |
| D_S3489 | T2 | D | Sweden | 4.Scand_Russia | endocervical | F | 2010 |  | ERS075212 |
| D_S3929 | T2 | D | Sweden | 4.Scand_Russia | endocervical | F | 2010 |  | ERS075201 |
| D_S4093 | T2 | D | Sweden | 4.Scand_Russia | endocervical | F | 2010 |  | ERS095074 |
| D_S4828 | T2 | D | Sweden | 4.Scand_Russia | endocervical | F | 2010 |  | ERS082976 |
| D_Soton128 | T1 | D | UK | 6.UK | endocervix | F | 2009 |  | ERS095096 |
| D_Soton15 | T1 | D | UK | 6.UK | endocervix | F | 2009 |  | ERS013807 |
| D_Soton150 | T2 | D | UK | 6.UK | endocervix | F | 2009 |  | ERS013823 |
| D_Soton42 | T2 | D | UK | 6.UK | endocervix | F | 2009 |  | ERS095089 |
| D_Soton47 | T2 | D | UK | 6.UK | endocervix | F | 2009 |  | ERS013812 |
| D_Soton49 | T1 | D | UK | 6.UK | endocervix | F | 2009 |  | ERS095091 |
| D_Soton54 | T2 | D | UK | 6.UK | endocervix | F | 2009 |  | ERS095092 |
| D_SotonD1 | T1 | D | UK | 6.UK | endocervix | F | 2009 | HE603229 | ERS008761 |
| D_SotonD2 | T2 | D | UK | 6.UK | endocervix | F | 2009 |  | ERS013784 |
| D_SotonD3 | T1 | D | UK | 6.UK | endocervix | F | 2009 |  | ERS013785 |
| D_SotonD4 | T2 | D | UK | 6.UK | endocervix | F | 2009 |  | ERS013786 |
| D_SotonD5 | T2 | D | UK | 6.UK | endocervix | F | 2009 | HE603230 | ERS013787 |
| D_SotonD6 | T2 | D | UK | 6.UK | endocervix | F | 2009 | HE603231 | ERS008762 |
| D_STN101 | T2 | D | UK | 6.UK | unk | unk | 1985 |  | ERS208565 |
| D_STN113 | T2 | D | UK | 6.UK | unk | unk | 1985 |  | ERS208567 |
| D_T9p | T1 | D | UK | 6.UK |  |  | 2012 |  | ERS095051 |
| D_UK466322 | T2 | D | UK | 6.UK | Cx/Urethral | F | 2012 |  | ERS208362 |
| D_UK663610 | T1 | D | UK | 6.UK | Cx/Urethral | F | 2012 |  | ERS208393 |
| D_UK750364 | T1 | D | UK | 6.UK | cervix | F | 2012 |  | ERS160254 |
| D_UK750376 | T2 | D | UK | 6.UK | urethral | M | 2012 |  | ERS160257 |
| D_UK750523 | T2 | D | UK | 6.UK | cervix | F | 2012 |  | ERS160260 |
| D_UK912432 | T2 | D | UK | 6.UK | Cx/Urethral | F | 2012 |  | ERS208400 |
| E_940U470 | T1 | E | USA | 7.N.America | urethra | M |  |  | ERS248053 |
| E_Ar152 | T1 | E | Argentina | 8.S.America | ocular | F | 2005 |  | ERS082953 |
| E_Ar182 | T1 | E | Argentina | 8.S.America | urethral | M | 2008 |  | ERS082955 |
| E_Ar250 | T1 | E | Argentina | 8.S.America | ocular | M | 2006 |  | ERS160297 |
| E_Ar427 | T1 | E | Argentina | 8.S.America | endocervical | F | 2011 |  | ERS082961 |
| E_Ar5 | T1 | E | Argentina | 8.S.America | endocervical | F | 2006 |  | ERS082951 |
| E_Ar7218 | T1 | E | Argentina | 8.S.America | ocular | M | 2004 |  | ERS082962 |
| E_Aus13 | T1 | E | Australia | 1.Aus | unk | unk |  |  | ERS153038 |
| E_Bour | T1 | E | USA | 7.N.America | ocular | M | 1959 | HE603212 | ERS001401 |
| E_C194 | T1 | E | UK | 6.UK | vaginal | F | 1993 |  | ERS177824 |
| E_C208 | T1 | E | UK | 6.UK | Urine | M | 2011 |  | ERS177826 |
| E_C236 | T1 | E | UK | 6.UK | Ur/Cx | F | 1971 |  | ERS177829 |
| E_C258 | T1 | E |  |  |  |  |  |  | ERS177832 |
| E_C37 | T1 | E | UK | 6.UK | Vaginal | F | 1985 |  | ERS177809 |
| E_C5 | T1 | E | UK | 6.UK | Vaginal | F | 1990 |  | ERS177804 |
| E_C58 | T1 | E | UK | 6.UK | Vaginal | F | 1992 |  | ERS177812 |
| E_CC15 | T1 | E | UK | 6.UK | cervical | F | 2009 |  | ERS095112 |
| E_CC35 | T1 | E | UK | 6.UK | urethral | M | 2010 |  | ERS095113 |
| E_DK20 | T1 | E | Denmark | 5.Europe | conjunctivitis |  | 1967 |  | ERS177774 |
| E_Fin127 | T1 | E | Finland | 4.Scand_Russia | endocervical | F | 2010 |  | ERS200063 |
| E_Fin129 | T1 | E | Finland | 4.Scand_Russia | endocervical | F | 2010 |  | ERS200064 |
| E_Fin142 | T1 | E | Finland | 4.Scand_Russia | endocervical | F | 2010 |  | ERS200065 |
| E_Fin155 | T1 | E | Finland | 4.Scand_Russia | endocervical | F | 2010 |  | ERS200070 |
| E_Fin159 | T1 | E | Finland | 4.Scand_Russia | endocervical | F | 2010 |  | ERS200116 |
| E_Fin172 | T1 | E | Finland | 4.Scand_Russia | endocervical | F | 2010 |  | ERS200074 |
| E_Fin184 | T1 | E | Finland | 4.Scand_Russia | endocervical | F | 2010 |  | ERS200077 |
| E_Fin185 | T1 | E | Finland | 4.Scand_Russia | endocervical | F | 2010 |  | ERS200078 |
| E_Fin194 | T1 | E | Finland | 4.Scand_Russia | endocervical | F | 2010 |  | ERS200081 |
| E_Fin198 | T1 | E | Finland | 4.Scand_Russia | endocervical | F | 2010 |  | ERS200082 |
| E_Fin214 | T1 | E | Finland | 4.Scand_Russia | endocervical | F | 2011 |  | ERS200087 |
| E_Fin220 | T1 | E | Finland | 4.Scand_Russia | endocervical | F | 2011 |  | ERS200090 |
| E_It246 | T1 | E | Italy | 5.Europe | endocervical | F | 2010 |  | ERS208511 |
| E_It363 | T1 | E | Italy | 5.Europe | endocervical | F | 2010 |  | ERS208510 |
| E_It769 | T1 | E | Italy | 5.Europe | endocervical | F | 2011 |  | ERS208512 |
| E_It807 | T1 | E | Italy | 5.Europe |  |  |  |  | ERS160294 |
| E_IU824 | T1 | E | USA | 7.N.America | endometrial | F |  |  | ERS177779 |
| E_IU888 | T1 | E | USA | 7.N.America | endometrial | F |  |  | ERS177780 |
| E_NL21 | T1 | E | Netherlands | 5.Europe | endocervical | F | 2001 |  | ERS161069 |
| E_NL23 | T1 | E | Netherlands | 5.Europe | endocervical | F | 2001 |  | ERS153033 |
| E_NL24 | T1 | E | Netherlands | 5.Europe | endocervical | F | 2001 |  | ERS153034 |
| E_NL26 | T1 | E | Netherlands | 5.Europe | endocervical | F | 2001 |  | ERS133274 |
| E_NL28 | T1 | E | Netherlands | 5.Europe | endocervical | F | 2001 |  | ERS133276 |
| E_NL29 | T1 | E | Netherlands | 5.Europe | endocervical | F | 2001 |  | ERS153035 |
| E_R1430 | T1 | E | Russia | 4.Scand_Russia | urethra | M | 2011 |  | ERS082938 |
| E_R16965 | T1 | E | Russia | 4.Scand_Russia | endocervical | F | 2011 |  | ERS095107 |
| E_R25114 | T1 | E | Russia | 4.Scand_Russia | endocervical | F | 2010 |  | ERS075219 |
| E_R26833 | T1 | E | Russia | 4.Scand_Russia | endocervical | F | 2010 |  | ERS082918 |
| E_R27091 | T1 | E | Russia | 4.Scand_Russia | cervical | F | 2010 |  | ERS075189 |
| E_R28017 | T1 | E | Russia | 4.Scand_Russia | cervical | F | 2010 |  | ERS075188 |
| E_R28044 | T1 | E | Russia | 4.Scand_Russia | endocervical | F | 2010 |  | ERS075217 |
| E_R29005 | T1 | E | Russia | 4.Scand_Russia | endocervical | F | 2010 |  | ERS082947 |
| E_R30444 | T1 | E | Russia | 4.Scand_Russia | endocervical | F | 2010 |  | ERS075223 |
| E_R32100 | T1 | E | Russia | 4.Scand_Russia | cervical | F | 2010 |  | ERS075186 |
| E_R33420 | T1 | E | Russia | 4.Scand_Russia | endocervical | F | 2010 |  | ERS133242 |
| E_R35067 | T1 | E | Russia | 4.Scand_Russia | urethra | M | 2010 |  | ERS075190 |
| E_R4159 | T1 | E | Russia | 4.Scand_Russia | urethra | M | 2011 |  | ERS082932 |
| E_R4195 | T1 | E | Russia | 4.Scand_Russia | endocervical | F | 2011 |  | ERS095066 |
| E_R4528 | T1 | E | Russia | 4.Scand_Russia | endocervical | F | 2011 |  | ERS095105 |
| E_R526 | T1 | E | Russia | 4.Scand_Russia | cervical | F | 2011 |  | ERS075191 |
| E_Rb10387 | T1 | E | UK | 6.UK | unspecified | M | 2010 |  | ERS017906 |
| E_Rb10392 | T1 | E | UK | 6.UK | cervix | F | 2010 |  | ERS177819 |
| E_Rb1392 | T1 | E | UK | 6.UK | cervix | F | 2010 |  | ERS066957 |
| E_S1019 | T1 | E | Sweden | 4.Scand_Russia | endocervical | F | 2010 |  | ERS095078 |
| E_S1086 | T1 | E | Sweden | 4.Scand_Russia | endocervical | F | 2010 |  | ERS075208 |
| E_S1148 | T1 | E | Sweden | 4.Scand_Russia | endocervical | F | 2010 |  | ERS095080 |
| E_S1227 | T1 | E | Sweden | 4.Scand_Russia | endocervical | F | 2010 |  | ERS095075 |
| E_S1528 | T1 | E | Sweden | 4.Scand_Russia | endocervical | F | 2010 |  | ERS095079 |
| E_S1613 | T1 | E | Sweden | 4.Scand_Russia | endocervical | F | 2010 |  | ERS095082 |
| E_S1618 | T1 | E | Sweden | 4.Scand_Russia | endocervical | F | 2010 |  | ERS082972 |
| E_S1886 | T1 | E | Sweden | 4.Scand_Russia | endocervical | F | 2010 |  | ERS082971 |
| E_S2384 | T1 | E | Sweden | 4.Scand_Russia | endocervical | F | 2010 |  | ERS095030 |
| E_S2491 | T1 | E | Sweden | 4.Scand_Russia | endocervical | F | 2010 |  | ERS082966 |
| E_S2699 | T1 | E | Sweden | 4.Scand_Russia | endocervical | F | 2011 |  | ERS095101 |
| E_S2713 | T1 | E | Sweden | 4.Scand_Russia | endocervical | F | 2011 |  | ERS095102 |
| E_S3024 | T1 | E | Sweden | 4.Scand_Russia | conjunctival | M | 2010 |  | ERS075202 |
| E_S3066 | T1 | E | Sweden | 4.Scand_Russia | endocervical | F | 2011 |  | ERS095111 |
| E_S3073 | T1 | E | Sweden | 4.Scand_Russia | endocervical | F | 2010 |  | ERS095071 |
| E_S3085 | T1 | E | Sweden | 4.Scand_Russia | endocervical | F | 2011 |  | ERS095103 |
| E_S3122 | T1 | E | Sweden | 4.Scand_Russia | endocervical | F | 2010 |  | ERS075205 |
| E_S3695 | T1 | E | Sweden | 4.Scand_Russia | endocervical | F | 2006 |  | ERS095085 |
| E_S3711 | T1 | E | Sweden | 4.Scand_Russia | endocervical | F | 2011 |  | ERS095104 |
| E_S3724 | T1 | E | Sweden | 4.Scand_Russia | endocervical | F | 2006 |  | ERS095086 |
| E_S3732 | T1 | E | Sweden | 4.Scand_Russia | unk | F | 2010 |  | ERS082964 |
| E_S4007 | T1 | E | Sweden | 4.Scand_Russia | endocervical | F | 2006 |  | ERS095087 |
| E_S4106 | T1 | E | Sweden | 4.Scand_Russia | endocervical | F | 2010 |  | ERS095073 |
| E_S4247 | T1 | E | Sweden | 4.Scand_Russia | urethra | M | 2010 |  | ERS082975 |
| E_S4324 | T1 | E | Sweden | 4.Scand_Russia | urethra | M | 2010 |  | ERS082970 |
| E_S4471 | T1 | E | Sweden | 4.Scand_Russia | endocervical | F | 2010 |  | ERS082967 |
| E_S581 | T1 | E | Sweden | 4.Scand_Russia | endocervical | F | 2011 |  | ERS153014 |
| E_Soton107 | T1 | E | UK | 6.UK | endocervix | F | 2009 |  | ERS075180 |
| E_Soton116 | T1 | E | UK | 6.UK | endocervix | F | 2009 |  | ERS075181 |
| E_Soton120 | T1 | E | UK | 6.UK | endocervix | F | 2009 |  | ERS075182 |
| E_Soton121 | T1 | E | UK | 6.UK | endocervix | F | 2009 |  | ERS013821 |
| E_Soton122 | T1 | E | UK | 6.UK | endocervix | F | 2009 |  | ERS013822 |
| E_Soton145 | T1 | E | UK | 6.UK | endocervix | F | 2009 |  | ERS075183 |
| E_Soton155 | T1 | E | UK | 6.UK | endocervix | F | 2009 |  | ERS013824 |
| E_Soton159 | T1 | E | UK | 6.UK | endocervix | F | 2009 |  | ERS075184 |
| E_Soton17 | T1 | E | UK | 6.UK | endocervix | F | 2009 |  | ERS075178 |
| E_Soton53 | T1 | E | UK | 6.UK | endocervix | F | 2009 |  | ERS075179 |
| E_Soton73 | T1 | E | UK | 6.UK | endocervix | F | 2009 |  | ERS013816 |
| E_Soton83 | T1 | E | UK | 6.UK | endocervix | F | 2009 |  | ERS095093 |
| E_SotonE1 | T1 | E | UK | 6.UK | endocervix | F | 2009 |  | ERS013788 |
| E_SotonE2 | T1 | E | UK | 6.UK | endocervix | F | 2009 |  | ERS013789 |
| E_SotonE3 | T1 | E | UK | 6.UK | endocervix | F | 2009 |  | ERS013790 |
| E_SotonE4 | T1 | E | UK | 6.UK | endocervix | F | 2009 | HE603232 | ERS013791 |
| E_SotonE5 | T1 | E | UK | 6.UK | endocervix | F | 2009 |  | ERS013792 |
| E_SotonE6 | T1 | E | UK | 6.UK | endocervix | F | 2009 |  | ERS013793 |
| E_SotonE7 | T1 | E | UK | 6.UK | endocervix | F | 2009 |  | ERS013794 |
| E_SotonE8 | T1 | E | UK | 6.UK | endocervix | F | 2009 | HE603233 | ERS008763 |
| E_SotonE9 | T1 | E | UK | 6.UK | endocervix | F | 2009 |  | ERS013795 |
| E_Sou102 | T1 | E | UK | 6.UK | unk | F | 1985 |  | ERS161060 |
| E_Sou60 | T1 | E | UK | 6.UK | unk | unk | 1985 |  | ERS161049 |
| E_Sou75 | T1 | E | UK | 6.UK | unk | unk | 1985 |  | ERS161054 |
| E_STN10 | T1 | E | UK | 6.UK | unk | unk | 1985 |  | ERS208555 |
| E_STN11 | T1 | E | UK | 6.UK | unk | unk | 1985 |  | ERS208556 |
| E_STN115 | T1 | E | UK | 6.UK | unk | unk | 1985 |  | ERS208568 |
| E_STN119 | T1 | E | UK | 6.UK | unk | unk | 1985 |  | ERS208569 |
| E_STN12 | T1 | E | UK | 6.UK | unk | unk | 1985 |  | ERS208557 |
| E_STN2 | T1 | E | UK | 6.UK | unk | unk | 1985 |  | ERS208554 |
| E_STN47 | T1 | E | UK | 6.UK | unk | unk | 1985 |  | ERS208562 |
| E_STN68 | T1 | E | UK | 6.UK | unk | unk | 1985 |  | ERS208563 |
| E_STN92 | T1 | E | UK | 6.UK | unk |  | 1985 |  | ERS208564 |
| E_SW2 | T1 | E | Sweden | 4.Scand_Russia | urethra | M | 2006 | FM865439 | ERS001397 |
| E_SW3 | T1 | E | Sweden | 4.Scand_Russia | cervix | F | 2001 | FM865440 | ERS001406 |
| E_Swab6 | T1 | E | UK | 6.UK | vaginal | F | 2010 |  | ERS177764 |
| E_SwabB4 | T1 | E | UK | 6.UK | Unk | F | 2010 |  | ERS015770 |
| E_UK220880 | T1 | E | UK | 6.UK | vaginal | F | 2012 |  | ERS160286 |
| E_UK34334 | T1 | E | UK | 6.UK | Urethral | M | 2012 |  | ERS208409 |
| E_UK466129 | T1 | E | UK | 6.UK | Cx/Urethral | F | 2012 |  | ERS208318 |
| E_UK466546 | T1 | E | UK | 6.UK | Cx/Urethral | F | 2012 |  | ERS208366 |
| E_UK582260 | T1 | E | UK | 6.UK | Urethral | M | 2012 |  | ERS208372 |
| E_UK582263 | T1 | E | UK | 6.UK | Cx/Urethral | F | 2012 |  | ERS208322 |
| E_UK583638 | T1 | E | UK | 6.UK | Urethral | M | 2012 |  | ERS208377 |
| E_UK584031 | T1 | E | UK | 6.UK | Cx/Urethral | F | 2012 |  | ERS208329 |
| E_UK663813 | T1 | E | UK | 6.UK | Cx/Urethral | F | 2012 |  | ERS208333 |
| E_UK663924 | T1 | E | UK | 6.UK | Cx/Urethral | F | 2012 |  | ERS208394 |
| E_UK663968 | T1 | E | UK | 6.UK | Urethral | M | 2012 |  | ERS208395 |
| E_UK664394 | T1 | E | UK | 6.UK | Urethral | M | 2012 |  | ERS208396 |
| E_UK769748 | T1 | E | UK | 6.UK | Cx/Urethral | F | 2012 |  | ERS208397 |
| E_UK769852 | T1 | E | UK | 6.UK | Urethral | M | 2012 |  | ERS208398 |
| E_UK912889 | T1 | E | UK | 6.UK | Cx/Urethral | F | 2012 |  | ERS208337 |
| E_UK913723 | T1 | E | UK | 6.UK | Cx/Urethral | F | 2012 |  | ERS208344 |
| E_UK913953 | T1 | E | UK | 6.UK | Vag | F | 2012 |  | ERS208346 |
| F_AddT9 | T1 | F | UK | 6.UK |  |  |  |  | ERS151259 |
| F_Aus20 | T1 | F | Australia | 1.Aus | unk | unk |  |  | ERS153031 |
| F_C55 | T1 | F | UK | 6.UK | Vaginal | F | 1987 |  | ERS177811 |
| F_Fin106 | T1 | F | Finland | 4.Scand_Russia | endocervical | F | 2010 |  | ERS200061 |
| F_Fin175 | T1 | F | Finland | 4.Scand_Russia | endocervical | F | 2010 |  | ERS200075 |
| F_Fin181 | T1 | F | Finland | 4.Scand_Russia | endocervical | F | 2010 |  | ERS200091 |
| F_Fin213 | T1 | F | Finland | 4.Scand_Russia | endocervical | F | 2011 |  | ERS200119 |
| F_Fin219 | T1 | F | Finland | 4.Scand_Russia | endocervical | F | 2011 |  | ERS200089 |
| F_HonMPB36 | T1 | F | Honduras | 8.S.America | cervical | F |  |  | ERS208517 |
| F_IC-Cal-3 | T1 | F | USA | 7.N.America | ocular (neonate) |  | 1960 |  | ERS200110 |
| F_It686 | T1 | F | Italy | 5.Europe | endocervical | F | 2011 |  | ERS208508 |
| F_It688 | T1 | F | Italy | 5.Europe | urethral | M | 2011 |  | ERS095034 |
| F_NI1 | T1 | F |  |  |  |  |  |  | ERS177836 |
| F_NL30 | T1 | F | Netherlands | 5.Europe | endocervical | F | 2001 |  | ERS133277 |
| F_NL31 | T1 | F | Netherlands | 5.Europe | endocervical | F | 2001 |  | ERS153039 |
| F_NL35 | T1 | F | Netherlands | 5.Europe | endocervical | F | 2001 |  | ERS161072 |
| F_NL36 | T1 | F | Netherlands | 5.Europe | endocervical | F | 2001 |  | ERS153003 |
| F_NL38 | T1 | F | Netherlands | 5.Europe | endocervical | F | 2001 |  | ERS200113 |
| F_R12921 | T1 | F | Russia | 4.Scand_Russia | endocervical | F | 2011 |  | ERS095069 |
| F_R28312 | T1 | F | Russia | 4.Scand_Russia | urethra | M | 2010 |  | ERS075220 |
| F_R4663 | T1 | F | Russia | 4.Scand_Russia | endocervical | F | 2011 |  | ERS095065 |
| F_R7369 | T1 | F | Russia | 4.Scand_Russia | endocervical | F | 2011 |  | ERS095068 |
| F_S1470 | T1 | F | Sweden | 4.Scand_Russia | endocervical | F | 2010 |  | ERS082974 |
| F_S1494 | T1 | F | Sweden | 4.Scand_Russia | endocervical | F | 2010 |  | ERS095081 |
| F_S2430 | T1 | F | Sweden | 4.Scand_Russia | urethra | M | 2010 |  | ERS075206 |
| F_S2526 | T1 | F | Sweden | 4.Scand_Russia | unk | F | 2010 |  | ERS075200 |
| F_S2595 | T1 | F | Sweden | 4.Scand_Russia | endocervical | F | 2010 |  | ERS082965 |
| F_S3948 | T1 | F | Sweden | 4.Scand_Russia | endocervical | F | 2010 |  | ERS095070 |
| F_S4410 | T1 | F | Sweden | 4.Scand_Russia | endocervical | F | 2010 |  | ERS075216 |
| F_Soton106 | T1 | F | UK | 6.UK | endocervix | F | 2009 |  | ERS095094 |
| F_Soton118 | T1 | F | UK | 6.UK | endocervix | F | 2009 |  | ERS095095 |
| F_Soton137 | T1 | F | UK | 6.UK | endocervix | F | 2009 |  | ERS095097 |
| F_Soton18 | T1 | F | UK | 6.UK | endocervix | F | 2009 |  | ERS013808 |
| F_Soton48 | T1 | F | UK | 6.UK | endocervix | F | 2009 |  | ERS095090 |
| F_Soton88 | T1 | F | UK | 6.UK | endocervix | F | 2009 |  | ERS013818 |
| F_SotonF1 | T1 | F | UK | 6.UK | endocervix | F | 2009 |  | ERS013796 |
| F_SotonF2 | T1 | F | UK | 6.UK | endocervix | F | 2009 |  | ERS013797 |
| F_SotonF3 | T1 | F | UK | 6.UK | endocervix | F | 2009 | HE603234 | ERS008764 |
| F_SotonF4 | T1 | F | UK | 6.UK | endocervix | F | 2009 |  | ERS013798 |
| F_Sou100 | T1 | F | UK | 6.UK | unk | M |  |  | ERS161059 |
| F_Sou87 | T1 | F | UK | 6.UK | unk |  | 1985 |  | ERS161055 |
| F_Sou89 | T1 | F | UK | 6.UK | unk | F |  |  | ERS161056 |
| F_STN110 | T1 | F | UK | 6.UK | unk | unk | 1985 |  | ERS208566 |
| F_STN15 | T1 | F | UK | 6.UK | unk | unk | 1985 |  | ERS208558 |
| F_STN22 | T1 | F | UK | 6.UK | unk | unk | 1985 |  | ERS208560 |
| F_SW4 | T1 | F | Sweden | 4.Scand_Russia | cervix | F | 2002 | FM865441 | ERS001414 |
| F_SW5 | T1 | F | Sweden | 4.Scand_Russia | cervix | F | 2002 | FM865442 | ERS001415 |
| F_Swab5 | T1 | F | UK | 6.UK | vaginal | F | 2010 |  | ERS013115 |
| F_SwabB1 | T1 | F | UK | 6.UK | vaginal | F | 2010 |  | ERS177788 |
| F_SwabB8 | T1 | F | UK | 6.UK | vaginal | F | 2010 |  | ERS015772 |
| F_UK220521 | T1 | F | UK | 6.UK | urethral | M | 2012 |  | ERS160285 |
| F_UK35155 | T1 | F | UK | 6.UK | Cx/Urethral | F | 2012 |  | ERS208353 |
| F_UK465966 | T1 | F | UK | 6.UK | Cx/Urethral | F | 2012 |  | ERS208359 |
| F_UK466273 | T1 | F | UK | 6.UK | Cx/Urethral | F | 2012 |  | ERS208319 |
| F_UK583012 | T1 | F | UK | 6.UK | Cx/Urethral | F | 2012 |  | ERS208383 |
| F_UK583072 | T1 | F | UK | 6.UK | Cx/Urethral | F | 2012 |  | ERS208379 |
| F_UK583468 | T1 | F | UK | 6.UK | Cx/Urethral | F | 2012 |  | ERS208376 |
| F_UK584026 | T1 | F | UK | 6.UK | Cx/Urethral | F | 2012 |  | ERS208374 |
| F_UK663442 | T1 | F | UK | 6.UK | Urethral | M | 2012 |  | ERS208391 |
| F_UK770010 | T1 | F | UK | 6.UK | Cx/Urethral | F | 2012 |  | ERS208399 |
| G_Ar112 | T2 | G | Argentina | 8.S.America | urethral | M | 2007 |  | ERS082952 |
| G_Ar246 | T2 | G | Argentina | 8.S.America | urethral | M | 2007 |  | ERS082958 |
| G_Aus1 | T2 | G | Australia | 1.Aus | unk | unk |  |  | ERS153018 |
| G_Aus16 | T2 | G | Australia | 1.Aus | unk | unk |  |  | ERS153028 |
| G_Aus17 | T2 | G | Australia | 1.Aus | unk | unk |  |  | ERS153029 |
| G_Aus18 | T2 | G | Australia | 1.Aus | unk | unk |  |  | ERS153036 |
| G_Aus19 | T2 | G | Australia | 1.Aus | unk | unk |  |  | ERS153037 |
| G_Fin144 | T2 | G | Finland | 4.Scand_Russia | endocervical | F | 2010 |  | ERS200066 |
| G_Fin153 | T2 | G | Finland | 4.Scand_Russia | endocervical | F | 2010 |  | ERS200069 |
| G_Fin158 | T2 | G | Finland | 4.Scand_Russia | endocervical | F | 2010 |  | ERS200071 |
| G_Fin205 | T2 | G | Finland | 4.Scand_Russia | endocervical | F | 2011 |  | ERS200117 |
| G_NL39 | T2 | G | Netherlands | 5.Europe | endocervical | F | 2001 |  | ERS153041 |
| G_NL40 | T2 | G | Netherlands | 5.Europe | endocervical | F | 2001 |  | ERS153006 |
| G_NL41 | T2 | G | Netherlands | 5.Europe | endocervical | F | 2001 |  | ERS161021 |
| G_NL42 | T2 | G | Netherlands | 5.Europe | endocervical | F | 2001 |  | ERS161022 |
| G_NL43 | T2 | G | Netherlands | 5.Europe | endocervical | F | 2001 |  | ERS153007 |
| G_NL44 | T2 | G | Netherlands | 5.Europe | endocervical | F | 2001 |  | ERS200143 |
| G_NL45 | T2 | G | Netherlands | 5.Europe | endocervical | F | 2001 |  | ERS153009 |
| G_NL46 | T2 | G | Netherlands | 5.Europe | endocervical | F | 2001 |  | ERS153010 |
| G_NL47 | T2 | G | Netherlands | 5.Europe | endocervical | F | 2001 |  | ERS161023 |
| G_NL48 | T2 | G | Netherlands | 5.Europe | endocervical | F | 2001 |  | ERS153011 |
| G_R15108 | T2 | G | Russia | 4.Scand_Russia | urethra | M | 2011 |  | ERS095026 |
| G_R2247 | T2 | G | Russia | 4.Scand_Russia | endocervical | F | 2011 |  | ERS082937 |
| G_R23736 | T2 | G | Russia | 4.Scand_Russia | endocervical | F | 2010 |  | ERS075218 |
| G_R27757 | T2 | G | Russia | 4.Scand_Russia | endocervical | F | 2010 |  | ERS082940 |
| G_R297 | T2 | G | Russia | 4.Scand_Russia | cervical | F | 2011 |  | ERS075199 |
| G_R3059 | T2 | G | Russia | 4.Scand_Russia | endocervical | F | 2011 |  | ERS082929 |
| G_R30591 | T2 | G | Russia | 4.Scand_Russia | cervical | F | 2010 |  | ERS075192 |
| G_R31458 | T2 | G | Russia | 4.Scand_Russia | urethra | M | 2010 |  | ERS082922 |
| G_R35506 | T2 | G | Russia | 4.Scand_Russia | endocervical | F | 2010 |  | ERS082927 |
| G_R36176 | T2 | G | Russia | 4.Scand_Russia | endocervical | F | 2010 |  | ERS082928 |
| G_R4175 | T2 | G | Russia | 4.Scand_Russia | endocervical | F | 2011 |  | ERS082931 |
| G_R459 | T2 | G | Russia | 4.Scand_Russia | endocervical | F | 2011 |  | ERS082934 |
| G_R9069 | T2 | G | Russia | 4.Scand_Russia | endocervical | F | 2011 |  | ERS082942 |
| G_R9892 | T2 | G | Russia | 4.Scand_Russia | urethra | M | 2011 |  | ERS082936 |
| G_S1471 | T2 | G | Sweden | 4.Scand_Russia | endocervical | F | 2010 |  | ERS082973 |
| G_S1824 | T2 | G | Sweden | 4.Scand_Russia | endocervical | F | 2010 |  | ERS075210 |
| G_S1846 | T2 | G | Sweden | 4.Scand_Russia | endocervical | F | 2010 |  | ERS095077 |
| G_S2477 | T2 | G | Sweden | 4.Scand_Russia | endocervical | F | 2010 |  | ERS095084 |
| G_S2956 | T2 | G | Sweden | 4.Scand_Russia | conjunctival | F | 2010 |  | ERS075204 |
| G_S3270 | T2 | G | Sweden | 4.Scand_Russia | endocervical | F | 2010 |  | ERS075211 |
| G_S3344 | T2 | G | Sweden | 4.Scand_Russia | endocervical | F | 2010 |  | ERS075214 |
| G_S4641 | T2 | G | Sweden | 4.Scand_Russia | endocervical | F | 2010 |  | ERS095083 |
| G_S4658 | T2 | G | Sweden | 4.Scand_Russia | conjunctival | M | 2010 |  | ERS082977 |
| G_Soton144 | T2 | G | UK | 6.UK | endocervix | F | 2009 |  | ERS095098 |
| G_Soton57 | T2 | G | UK | 6.UK | endocervix | F | 2009 |  | ERS013813 |
| G_SotonG1 | T2 | G | UK | 6.UK | endocervix | F | 2009 | HE603235 | ERS013800 |
| G_SotonG2 | T2 | G | UK | 6.UK | endocervix | F | 2009 |  | ERS013801 |
| G_SotonG3 | T2 | G | UK | 6.UK | endocervix | F | 2009 |  | ERS013802 |
| G_SotonG4 | T2 | G | UK | 6.UK | endocervix | F | 2009 |  | ERS013803 |
| G_UK221409 | T2 | G | UK | 6.UK |  |  |  |  | ERS160283 |
| G_UK582500 | T2 | G | UK | 6.UK | Urethral | M | 2012 |  | ERS208385 |
| G_UK750369 | T2 | G | UK | 6.UK | cervix | F | 2012 |  | ERS160255 |
| G_UK913362 | T2 | G | UK | 6.UK | Cx/Urethral | F | 2012 |  | ERS208404 |
| G_UW57 | T2 | G | USA | 7.N.America | cervix |  | 1971 |  | ERS133245 |
| H_Fin109 | T2 | H | Finland | 4.Scand_Russia | endocervical | F | 2010 |  | ERS200142 |
| H_NL49 | T2 | H | Netherlands | 5.Europe | endocervical | F | 2001 |  | ERS161024 |
| H_NL50 | T2 | H | Netherlands | 5.Europe | endocervical | F | 2001 |  | ERS161025 |
| H_NL51 | T2 | H | Netherlands | 5.Europe | endocervical | F | 2001 |  | ERS161026 |
| H_NL53 | T2 | H | Netherlands | 5.Europe | endocervical | F | 2001 |  | ERS161028 |
| H_NL54 | T2 | H | Netherlands | 5.Europe | endocervical | F | 2001 |  | ERS161029 |
| H_NL56 | T2 | H | Netherlands | 5.Europe | endocervical | F | 2001 |  | ERS161031 |
| H_R13670 | T2 | H | Russia | 4.Scand_Russia | urethral | M | 2011 |  | ERS133241 |
| H_R25308 | T2 | H | Russia | 4.Scand_Russia | endocervical | F | 2010 |  | ERS082933 |
| H_R27887 | T2 | H | Russia | 4.Scand_Russia | endocervical | F | 2010 |  | ERS075222 |
| H_R31975 | T2 | H | Russia | 4.Scand_Russia | endocervical | F | 2010 |  | ERS082923 |
| H_S1026 | T2 | H | Sweden | 4.Scand_Russia | endocervical | F | 2010 |  | ERS082983 |
| H_S1314 | T2 | H | Sweden | 4.Scand_Russia | endocervical | F | 2010 |  | ERS082978 |
| H_S1432 | T2 | H | Sweden | 4.Scand_Russia | endocervical | F | 2010 |  | ERS082985 |
| H_S269 | T2 | H | Sweden | 4.Scand_Russia | unk | F | 2011 |  | ERS133233 |
| H_S4377 | T2 | H | Sweden | 4.Scand_Russia | endocervical | F | 2010 |  | ERS133235 |
| H_UW4 | T2 | H | USA | 7.N.America | cervix | F | 1965 |  | ERS177776 |
| H_UW43 | T2 | H | USA | 7.N.America | cervix | F | 1971 |  | ERS200111 |
| I_NL58 | T2 | I-Ia | Netherlands | 5.Europe | endocervical | F | 2001 |  | ERS161032 |
| I_NL63 | T2 | I-Ia | Netherlands | 5.Europe | endocervical | F | 2001 |  | ERS161035 |
| I_NL66 | T2 | I-Ia | Netherlands | 5.Europe | endocervical | F | 2001 |  | ERS161076 |
| I_NL67 | T2 | I-Ia | Netherlands | 5.Europe | endocervical | F | 2001 |  | ERS161036 |
| I_NL69 | T2 | I-Ia | Netherlands | 5.Europe | endocervical | F | 2001 |  | ERS161073 |
| I_NL70 | T2 | I-Ia | Netherlands | 5.Europe | endocervical | F | 2001 |  | ERS161037 |
| I_NL72 | T2 | I-Ia | Netherlands | 5.Europe | endocervical | F | 2001 |  | ERS161077 |
| I_S2459 | T2 | I-Ia | Sweden | 4.Scand_Russia | endocervical | F | 2010 |  | ERS075215 |
| I_Soton103 | T2 | I-Ia | UK | 6.UK | endocervix | F | 2009 |  | ERS013820 |
| I_Soton34 | T2 | I-Ia | UK | 6.UK | endocervix | F | 2009 |  | ERS013810 |
| I_UK913341 | T2 | I-Ia | UK | 6.UK | Vag | F | 2012 |  | ERS208340 |
| I_UW12 | T2 | I-Ia | USA | 7.N.America | urethra |  | 1966 |  | ERS200144 |
| Ia_SotonIa1 | T2 | I-Ia | UK | 6.UK | endocervix | F | 2009 | HE603236 | ERS013804 |
| Ia_SotonIa3 | T2 | I-Ia | UK | 6.UK | endocervix | F | 2009 | HE603237 | ERS013805 |
| J_C114 | T1 | J | UK | 6.UK | Vaginal | F | 1993 |  | ERS177815 |
| J_NL55 | T1 | J | Netherlands | 5.Europe | endocervical | F | 2001 |  | ERS161030 |
| J_NL76 | T2 | J | Netherlands | 5.Europe | endocervical | F | 2001 |  | ERS161039 |
| J_NL78 | T2 | J | Netherlands | 5.Europe | endocervical | F | 2001 |  | ERS161040 |
| J_S1254 | T2 | J | Sweden | 4.Scand_Russia | endocervical | F | 2010 |  | ERS082984 |
| J_S178 | T2 | J | Sweden | 4.Scand_Russia | endocervical | F | 2011 |  | ERS095099 |
| J_S3107 | T2 | J | Sweden | 4.Scand_Russia | endocervical | F | 2010 |  | ERS082981 |
| J_S42 | T2 | J | Sweden | 4.Scand_Russia | endocervical | F | 2011 |  | ERS095088 |
| J_S4281 | T2 | J | Sweden | 4.Scand_Russia | endocervical | F | 2010 |  | ERS082982 |
| J_S4821 | T2 | J | Sweden | 4.Scand_Russia | endocervical | F | 2010 |  | ERS133236 |
| J_Soton72 | T2 | J | UK | 6.UK | endocervix | F | 2009 |  | ERS013815 |
| J_Soton98 | T2 | J | UK | 6.UK | endocervix | F | 2009 |  | ERS013819 |
| J_Sou106 | T1 | J | UK | 6.UK | unk | F | 1985 |  | ERS161063 |
| J_UK35672 | T2 | J | UK | 6.UK | Urethral | M | 2012 |  | ERS208412 |
| J_UK583546 | T2 | J | UK | 6.UK | Urethral | M | 2012 |  | ERS208375 |
| J_UK583676 | T2 | J | UK | 6.UK | Urethral | M | 2012 |  | ERS208384 |
| J_UK913454 | T2 | J | UK | 6.UK | Urethral | M | 2012 |  | ERS208406 |
| J_UW36 | T2 | J | USA | 7.N.America | cervix | F | 1971 |  | ERS177783 |
| K_Ar650 | T2 | K | Argentina | 8.S.America | urethral | M | 2006 |  | ERS208546 |
| K_Ar74 | T2 | K | Argentina | 8.S.America | ocular | F | 2005 |  | ERS208545 |
| K_Fin128 | T2 | K | Finland | 4.Scand_Russia | endocervical | F | 2010 |  | ERS200115 |
| K_Fin139 | T2 | K | Finland | 4.Scand_Russia | endocervical | F | 2010 |  | ERS200092 |
| K_Fin202 | T2 | K | Finland | 4.Scand_Russia | endocervical | F | 2011 |  | ERS200093 |
| K_Fin204 | T2 | K | Finland | 4.Scand_Russia | endocervical | F | 2011 |  | ERS200094 |
| K_NL81 | T2 | K | Netherlands | 5.Europe | endocervical | F | 2001 |  | ERS161078 |
| K_NL82 | T2 | K | Netherlands | 5.Europe | endocervical | F | 2001 |  | ERS161074 |
| K_NL83 | T2 | K | Netherlands | 5.Europe | endocervical | F | 2001 |  | ERS161079 |
| K_NL84 | T2 | K | Netherlands | 5.Europe | endocervical | F | 2001 |  | ERS161075 |
| K_NL85 | T2 | K | Netherlands | 5.Europe | endocervical | F | 2001 |  | ERS161041 |
| K_NL87 | T2 | K | Netherlands | 5.Europe | endocervical | F | 2001 |  | ERS161080 |
| K_R11642 | T2 | K | Russia | 4.Scand_Russia | endocervical | F | 2011 |  | ERS095109 |
| K_R13207 | T2 | K | Russia | 4.Scand_Russia | endocervical | F | 2011 |  | ERS095106 |
| K_R14876 | T2 | K | Russia | 4.Scand_Russia | urethra | M | 2011 |  | ERS095108 |
| K_R15212 | T2 | K | Russia | 4.Scand_Russia | endocervical | F | 2011 |  | ERS095110 |
| K_R2084 | T2 | K | Russia | 4.Scand_Russia | endocervical | F | 2011 |  | ERS082939 |
| K_R26881 | T2 | K | Russia | 4.Scand_Russia | endocervical | F | 2010 |  | ERS082943 |
| K_R27128 | T2 | K | Russia | 4.Scand_Russia | endocervical | F | 2010 |  | ERS082946 |
| K_R32840 | T2 | K | Russia | 4.Scand_Russia | endocervical | F | 2010 |  | ERS082944 |
| K_R34345 | T2 | K | Russia | 4.Scand_Russia | endocervical | F | 2010 |  | ERS082926 |
| K_R34962 | T2 | K | Russia | 4.Scand_Russia | urethra | M | 2010 |  | ERS082925 |
| K_S143 | T2 | K | Sweden | 4.Scand_Russia | endocervical | F | 2011 |  | ERS082986 |
| K_S4034 | T2 | K | Sweden | 4.Scand_Russia | endocervical | F | 2010 |  | ERS133234 |
| K_S4229 | T2 | K | Sweden | 4.Scand_Russia | urethra | F | 2010 |  | ERS075207 |
| K_Soton13 | T2 | K | UK | 6.UK | endocervix | F | 2009 |  | ERS013806 |
| K_Soton32 | T2 | K | UK | 6.UK | endocervix | F | 2009 |  | ERS013809 |
| K_Soton37 | T2 | K | UK | 6.UK | endocervix | F | 2009 |  | ERS013811 |
| K_Soton63 | T2 | K | UK | 6.UK | endocervix | F | 2009 |  | ERS013814 |
| K_SotonK1 | T2 | K | UK | 6.UK | endocervix | F | 2009 | HE603238 | ERS013799 |
| K_UK582774 | T2 | K | UK | 6.UK | Cx/Urethral | F | 2012 |  | ERS208380 |
| K_UK583237 | T2 | K | UK | 6.UK | Cx/Urethral | F | 2012 |  | ERS208327 |
| K_UK663060 | T2 | K | UK | 6.UK | Cx/Urethral | F | 2012 |  | ERS208389 |
| K_UK663124 | T2 | K | UK | 6.UK | Cx/Urethral | F | 2012 |  | ERS208387 |
| K_Ur769079 | T2 | K | UK | 6.UK | Urine | F | 2012 |  | ERS208541 |
| K_UW31 | T2 | K | USA | 7.N.America | cervix | F | 1973 |  | ERS177784 |
| L1_115 | LGV | L1 | S_Africa | 3.Africa | unk |  |  | HE603218 | ERS001411 |
| L1_224 | LGV | L1 | S_Africa | 3.Africa | unk |  |  | HE603220 | ERS001404 |
| L1_440 | LGV | L1 | USA | 7.N.America | lymph node | M | 1968 |  | ERS001396 |
| L1_L1034 | LGV | L1 | S_Africa | 3.Africa | urethral | M | 1994 |  | ERS161085 |
| L1_L115p10 | LGV | L1 | S_Africa | 3.Africa | urethral | M | 1986 |  | ERS161112 |
| L1_L146 | LGV | L1 | S_Africa | 3.Africa | ulcer | M | 1986 |  | ERS161094 |
| L1_L165 | LGV | L1 | S_Africa | 3.Africa | ulcer | M | 1986 |  | ERS161095 |
| L1_L224 | LGV | L1 | S_Africa | 3.Africa | urethral | M | 1986 |  | ERS161108 |
| L1_L232 | LGV | L1 | S_Africa | 3.Africa | urethral | M | 1987 |  | ERS161109 |
| L1_L246 | LGV | L1 | S_Africa | 3.Africa | urethral | M | 1987 |  | ERS161096 |
| L1_L82 | LGV | L1 | S_Africa | 3.Africa | urethral | M | 1985 |  | ERS161107 |
| L1_L867 | LGV | L1 | S_Africa | 3.Africa | urethral | M | 1993 |  | ERS161110 |
| L1_L942 | LGV | L1 | S_Africa | 3.Africa | urethral | M | 1994 |  | ERS161111 |
| L1_LGV913 | LGV | L1 | S_Africa | 3.Africa |  | M |  |  | ERS066954 |
| L1_LGV98 | LGV | L1 | S_Africa | 3.Africa |  | M |  |  | ERS066953 |
| L1_SA16 | LGV | L1 | S_Africa | 3.Africa | genital ulcer | M | 1995 | HE603219 | ERS003316 |
| L1_SA160 | LGV | L1 | S_Africa | 3.Africa | ulcer | M | 1986 |  | ERS208531 |
| L1_SA409 | LGV | L1 | S_Africa | 3.Africa | ulcer | M | 1990 |  | ERS208533 |
| L1_SABY216 | LGV | L1 | S_Africa | 3.Africa | ulcer | M | 1999 |  | ERS208529 |
| L1_Ur583800 | LGV | L1 | UK | 6.UK | Urine | M | 2012 |  | ERS208537 |
| L2_470LN870 | LGV | L2 | USA | 7.N.America | lymph node | M | 1968 |  | ERS248055 |
| L2_514BU11 | LGV | L2 | USA | 7.N.America | bubo | M | 1968 |  | ERS248052 |
| L2_526BU5 | LGV | L2 | USA | 7.N.America | bubo | M | 1968 |  | ERS373086 |
| L2_L198 | LGV | L2 | S_Africa | 3.Africa | ulcer | M | 1986 |  | ERS161100 |
| L2_L694 | LGV | L2 | S_Africa | 3.Africa | urethral | M | 1993 |  | ERS161114 |
| L2_LGV173 | LGV | L2 | S_Africa | 3.Africa |  | M |  |  | ERS066952 |
| L2_SF25667 | LGV | L2 | USA | 7.N.America | rectal | M | 1981 |  | ERS248067 |
| L2_SF40369 | LGV | L2 | USA | 7.N.America | rectal | M | 1984 |  | ERS248047 |
| L2_SFUW396 | LGV | L2 | USA | 7.N.America | rectal | M | 1980s |  | ERS248050 |
| L2b_795 | LGV | L2b | France | 5.Europe | rectum | M | 2004 | HE603221 | ERS001409 |
| L2b_8200 | LGV | L2b | Sweden | 4.Scand_Russia | proctitis | M | 2007 | HE603222 | ERS004108 |
| L2b_C1 | LGV | L2b | Canada | 7.N.America | rectum | M | 2004 | HE603223 | ERS001398 |
| L2b_C2 | LGV | L2b | Canada | 7.N.America | rectum | M | 2005 | HE603224 | ERS001399 |
| L2b_CV204 | LGV | L2b | France | 5.Europe | rectum | M | 2006 | HE603225 | ERS003307 |
| L2b_H17IMS | LGV | L2b | UK | 6.UK | rectal | M | 2008 |  | ERS095036 |
| L2b_HPA1 | LGV | L2b | UK | 6.UK | rectal swab | M | 2005 |  | ERS133246 |
| L2b_HPA21 | LGV | L2b | UK | 6.UK | rectal | M | 2009 |  | ERS095037 |
| L2b_HPA27 | LGV | L2b | UK | 6.UK | rectal swab | M | 2005 |  | ERS133248 |
| L2b_HPA29 | LGV | L2b | UK | 6.UK | rectal swab | M | 2004 |  | ERS133249 |
| L2b_HPA31 | LGV | L2b | UK | 6.UK | rectal swab | M | 2005 |  | ERS133250 |
| L2b_HPA34 | LGV | L2b | UK | 6.UK | rectal swab | M | 2008 |  | ERS133252 |
| L2b_LST | LGV | L2b | France | 5.Europe | rectum | M | 2008 | HE603226 | ERS003315 |
| L2b_s11 | LGV | L2b | Netherlands | 5.Europe | penile ulcer | M | 2004 | HE603213 | ERS001408 |
| L2b_s121 | LGV | L2b | Netherlands | 5.Europe | anal swab | M | 2005 | HE603216 | ERS001402 |
| L2b_s300 | LGV | L2b | Netherlands | 5.Europe | anal swab | M | 2004 | HE603217 | ERS001413 |
| L2b_s750 | LGV | L2b | Netherlands | 5.Europe | anal swab | M | 2004 | HE603215 | ERS001410 |
| L2b_s906 | LGV | L2b | Netherlands | 5.Europe | anal swab | M | 2005 | HE603214 | ERS001395 |
| L2b_SF156531 | LGV | L2b | USA | 7.N.America | rectal | M | 2001 |  | ERS373084 |
| L2b_SF156710 | LGV | L2b | USA | 7.N.America | rectal | M | 2003 |  | ERS373085 |
| L2b_SF156740 | LGV | L2b | USA | 7.N.America | rectal | M | 2003 |  | ERS248080 |
| L2b_SF41806 | LGV | L2b | USA | 7.N.America | rectal | M | 1984 |  | ERS248048 |
| L2b_SF46445 | LGV | L2b | USA | 7.N.America | rectal | M | 1985 |  | ERS248049 |
| L2b_UCH1 | LGV | L2b | UK | 6.UK | proctitis | M | 2006 | AM886279 | ERS001407 |
| L2b_UCH2 | LGV | L2b | UK | 6.UK | proctitis |  |  | HE603227 | ERS001405 |
| L3_404 | LGV | L3 | USA | 7.N.America | lymph node | M | 1967 | HE603228 | ERS001416 |
