# Supplementary material for: The Nature and Extent of Plasmid Variation in Chlamydia trachomatis
Source: Microorganisms. 2020 Mar 6;8(3):373. doi: 10.3390/microorganisms8030373 (PMC7143637; doi:10.3390/microorganisms8030373)
Supplement: Supplementary file 1 [file microorganisms-08-00373-s001.zip › Jones et al Supplementary Data Files/Table S2.docx]

Supplementary data table 2: Summary tables of raw data for each *C. trachomatis* plasmid CDS

| **CDS1** | |  |  |  |  |  |  |  |  |
| --- | --- | --- | --- | --- | --- | --- | --- | --- | --- |
| Base | Number of SNPs | Number of SNPs in tri-allelic positions | P.Mutant code | SNP Change | Pre-AA | Post-AA | Synonmous/Non-Synonmous SNP | Polar / Non Polar / Basic / Acidic | Codon Base Change |
| 190 | 1 |  | AGT | AAT | S | N | Non-Synonymous | p-p | 2 |
| 194 | 55 |  | AGA | AGG | R | R | Synonymous |  | 3 |
| 200 | 281 |  | TTT | TTC | F | F | Synonymous |  | 3 |
| 213 | 1 |  | GAC | AAC | D | N | Non-Synonymous | A-P | 1 |
| 246 | 284 |  | TAT | CAT | Y | H | Non-Synonymous | P-B | 1 |
| 249 | 2 |  | CTA | TTA | L | L | Synonymous |  | 1 |
| 288 | 2 |  | GTT | ATT | V | I | Non-Synonymous | NP-NP | 1 |
| 314 | 33 |  | TCG | TCA | S | S | Synonymous |  | 3 |
| 337 | 2 |  | TCT | TAT | S | Y | Non-Synonymous | P-P | 2 |
| 398 | 284 |  | GTT | GTC | V | V | Synonymous |  | 3 |
| 410 | 285 |  | GCA | GCC | A | A | Synonymous |  | 3 |
| 437 | 5 |  | TAC | TAT | Y | Y | Synonymous |  | 3 |
| 591 | 1 |  | GCG | TCG | A | S | Non-Synonymous | NP-P | 1 |
| 699 | 2 | 1 | GCA | ACA | A | T | Non-Synonymous | NP-P | 1 |
|  |  | 1 | GCA | TCA | A | S | Non-Synonymous | NP-P | 1 |
| 750 | 1 |  | AAA | CAA | K | Q | Non-Synonymous | B-P | 1 |
| 785 | 55 |  | GAA | GAG | E | E | Synonymous |  | 3 |
| 805 | 4 |  | TGT | TAT | C | Y | Non-Synonymous | P-P | 2 |
| 822 | 30 |  | GTA | ATA | V | I | Non-Synonymous | NP-NP | 1 |
| 894 | 55 |  | CAT | TAT | H | Y | Non-Synonymous | b-p | 1 |
| 910 | 9 |  | ATA | AAA | I | K | Non-Synonymous | NP-B | 1 |
| 923 | 54 |  | CCC | CCT | P | P | Synonymous |  | 3 |
| 951 | 18 |  | CAT | TAT | H | Y | Non-Synonymous | B-P | 1 |
| 965 | 1 |  | ATA | ATG | I | M | Non-Synonymous | NP-NP | 3 |
| 1004 | 196 |  | CTT | CTC | L | L | Synonymous |  | 3 |
| 1050 | 1 |  | ATT | GTT | I | G | Non-Synonymous | NP-P | 1 |
| 1051 | 285 |  | ATT | AGT | I | M | Non-Synonymous | NP-NP | 2 |
| 1068 | 2 |  | CCC | TCC | P | S | Non-Synonymous | NP-P | 1 |
| 1071 | 55 |  | ACA | CCA | T | P | Non-Synonymous | p-mp | 1 |
| 1076 | 1 |  | ATA | ATG | I | M | Non-Synonymous | NP-NP | 3 |
| 1080 | 7 |  | TGA | GGA | STOP | G | Non-Synonymous | STOP-P | 1 |

| **CDS2** | |  |  |  |  |  |  |  |  |
| --- | --- | --- | --- | --- | --- | --- | --- | --- | --- |
| Base | Number of SNPs | Number of SNPs in tri-allelic positions | P.Mutant code | SNP Change | Pre-AA | Post-AA | Synonmous/Non-Synonmous SNP | Polar / Non Polar / Basic / Acidic | Codon Base Change |
| 1139 | 1 |  | ATC | GTC | L | L | Synonymous |  | 3 |
| 1146 | 34 |  | GTA | GCA | M | T | Non-Synonymous | NP-P | 2 |
| 1147 | 125 |  | GTA | GTC | M | V | Non_Synonymous | NP-NP | 1 |
| 1151 | 1 |  | GAA | AAA | K | K | Synonymous |  | 3 |
| 1175 | 194 |  | TCT | CCT | S | S | Synonymous |  | 3 |
| 1178 | 1 |  | AAA | GAA | R | S | Non_Synonymous | B-P | 3 |
| 1217 | 195 |  | ACA | GCA | T | K | Non-Synonymous | P-B | 3 |
| 1223 | 4 |  | GAA | AAA | K | K | Synonymous |  | 3 |
| 1301 | 282 |  | AAA | GAA | K | K | Synonymous |  | 3 |
| 1376 | 2 |  | CCT | ACT | S | S | Synonymous |  | 3 |
| 1461 | 3 |  | TTA | TCA | I | T | Non_Synonymous | NP-P | 2 |
| 1565 | 9 |  | TGG | CGG | G | G | Synonymous |  | 3 |
| 1607 | 196 |  | TAA | CAA | N | N | Synonymous |  | 3 |
| 1637 | 55 |  | ACG | GCG | A | A | Synonymous |  | 3 |
| 1651 | 2 |  | TGA | TGT | S | C | Non_Synonymous | P-P | 1 |
| 1664 | 55 |  | GCA | ACA | T | T | Synonymous |  | 3 |
| 1698 | 1 |  | AGC | ACC | R | P | Non_Synonymous | B-P | 2 |
| 1731 | 1 |  | TCG | TAG | A | D | Non-Synonymous | NP-A | 2 |
| 1732 | 1 |  | TCG | TCA | A | T | Non-Synonymous | NP-P | 1 |
| 1817 | 33 |  | TCT | CCT | S | S | Synonymous |  | 3 |
| 1826 | 1 |  | CCG | TCG | A | A | Synonymous |  | 3 |
| 1828 | 1 |  | CCG | CCA | A | T | Non_Synonymous | NP-P | 1 |
| 1856 | 55 |  | CTA | TTA | I | I | Synonymous |  | 3 |
| 1904 | 195 |  | CCT | TCT | S | S | Synonymous |  | 3 |
| 2036 | 1 |  | AAG | GAG | E | E | Synonymous |  | 3 |
| 2075 | 286 |  | TGA | CGA | S | S | Synonymous |  | 3 |

|  | |  | |  | |  | |  | | |  |  | |  | |  | |  | |
| --- | --- | --- | --- | --- | --- | --- | --- | --- | --- | --- | --- | --- | --- | --- | --- | --- | --- | --- | --- |
| **CDS3** | |  | |  | |  | |  | | |  |  | |  | |  | |  | |
| Base | | Number of SNPs | | Number of SNPs in tri-allelic positions | | P.Mutant code | | SNP Change | | | Pre-AA | Post-AA | | Synonmous/ Non-Synonmous SNP | | Polar / Non Polar / Basic / Acidic | | Codon Base Change | |
| 2399 | | 195 | |  | | TCT | | TCC | | | S | S | | Synonymous | |  | | 3 | |
| 2432 | | 2 | |  | | TAC | | TAT | | | Y | Y | | Synonymous | |  | | 3 | |
| 2505 | | 1 | |  | | ATG | | GTG | | | M | V | | Non-Synonymous | | NP-NP | | 1 | |
| 2583 | | 1 | |  | | TTC | | CTC | | | F | L | | Non-Synonymous | | NP-NP | | 1 | |
| 2627 | | 195 | |  | | AGT | | AGC | | | S | S | | Synonymous | |  | | 3 | |
| 2720 | | 55 | |  | | GAT | | GAC | | | D | D | | Synonymous | |  | | 3 | |
| 2731 | | 1 | |  | | GCA | | GAA | | | A | E | | Non-Synonymous | | NP-A | | 2 | |
| 2760 | | 1 | |  | | GGC | | AGC | | | G | S | | Non-Synonymous | | P-P | | 1 | |
| 2766 | | 1 | |  | | TCT | | CCT | | | S | P | | Non-Synonymous | | P-NP | | 1 | |
| 2830 | | 284 | |  | | GTA | | GCA | | | V | A | | Non-Synonymous | | NP-NP | | 2 | |
| 2832 | | 54 | |  | | GCG | | ACG | | | A | T | | Non-Synonymous | | NP-P | | 1 | |
| 2858 | | 5 | |  | | TTC | | TTA | | | F | L | | Non-Synonymous | | NP-NP | | 3 | |
| 2865 | | 1 | |  | | GGC | | TGC | | | G | C | | Non-Synonymous | | P-P | | 1 | |
| 2866 | | 54 | |  | | GGC | | GCC | | | G | A | | Non-Synonymous | | P-NP | | 2 | |
| 2943 | | 2 | |  | | AGA | | CGA | | | R | R | | Synonymous | |  | | 1 | |
| 2945 | | 287 | |  | | AGA | | AGG | | | R | R | | Synonymous | |  | | 3 | |
| 2963 | | 55 | |  | | ACA | | ACT | | | T | T | | Synonymous | |  | | 3 | |
| 3023 | | 1 | |  | | TTC | | TTT | | | F | F | | Synonymous | |  | | 3 | |
| 3062 | | 3 | |  | | CGG | | CGA | | | R | R | | Synonymous | |  | | 3 | |
| 3066 | | 100 | |  | | ATT | | GTT | | | I | V | | Non-Synonymous | | NP-NP | | 1 | |
| 3111 | | 1 | |  | | GAT | | AAT | | | D | N | | Non-Synonymous | | A-P | | 1 | |
| 3140 | | 162 | |  | | GTA | | GTG | | | V | V | | Synonymous | |  | | 3 | |
| 3145 | | 7 | |  | | GTA | | GTG | | | V | V | | Synonymous | |  | | 3 | |
| 3158 | | 285 | |  | | ACG | | ACA | | | T | T | | Synonymous | |  | | 3 | |
| 3188 | | 4 | | 2 | | AGT | | AGC | | | S | S | | Synonymous | |  | | 3 | |
|  |  |  |  | 2 | | AGT | | AGA | | | S | T | | Non-Synonymous | | P-P | | 3 | |
| 3209 | | 283 | |  | | AAC | | AAT | | | N | N | | Synonymous | |  | | 3 | |
| 3217 | | 2 | |  | | GCG | | GAG | | | A | E | | Non-Synonymous | | NP-A | | 2 | |
| 3229 | | 61 | |  | | CGG | | CAG | | | R | Q | | Non-Synonymous | | B-P | | 2 | |
| 3259 | | 1 | |  | | GTA | | GCA | | | V | A | | Non-Synonymous | | NP-NP | | 2 | |
| 3383 | | 5 | |  | | TGT | | TGC | | | C | C | | Synonymous | |  | | 3 | |
| 3393 | | 2 | |  | | CTA | | TTA | | | L | L | | Synonymous | |  | | 1 | |
| 3508 | | 5 | |  | | TCG | | TTG | | | S | L | | Non-Synonymous | | 2 | | 2 | |
| **CDS4** |  | |  | |  | |  | |  |  | | |  | |  | |  | |  |
| Base | Number of SNPs | | Number of SNPs in tri-allelic positions | | P.Mutant code | | SNP Change | | Pre-AA | Post-AA | | | Synonmous/ Non-Synonmous SNP | | Polar / Non Polar / Basic / Acidic | | Codon Base Change | |  |
| 3802 | 55 | |  | | TTA | | TTG | | L | L | | | Synonymous | |  | | 3 | |  |
| 3808 | 85 | |  | | ACC | | ACT | | T | T | | | Synonymous | |  | | 3 | |  |
| 3842 | 52 | |  | | GTT | | ATT | | V | I | | | Non-Synonymous | | NP-NP | | 1 | |  |
| 3916 | 26 | |  | | TTC | | TTT | | F | F | | | Synonymous | |  | | 3 | |  |
| 3919 | 1 | |  | | TTA | | TTG | | L | L | | | Synonymous | |  | | 3 | |  |
| 3940 | 5 | |  | | CGG | | CGA | | R | R | | | Synonymous | |  | | 3 | |  |
| 4006 | 161 | |  | | TTA | | TTG | | L | L | | | Synonymous | |  | | 3 | |  |
| 4184 | 274 | |  | | CCT | | TCT | | P | S | | | Non-Synonymous | | NP-P | | 1 | |  |
| 4204 | 132 | |  | | GGG | | GGA | | G | G | | | Synonymous | |  | | 3 | |  |
| 4297 | 55 | |  | | CGT | | CGC | | R | R | | | Synonymous | |  | | 3 | |  |
| 4303 | 2 | |  | | CCA | | CCG | | P | P | | | Synonymous | |  | | 3 | |  |
| 4306 | 164 | |  | | AAT | | AAC | | N | N | | | Synonymous | |  | | 3 | |  |
| 4408 | 55 | |  | | TTA | | TTC | | L | F | | | Non-Synonymous | | NP-NP | | 3 | |  |
| 4417 | 2 | |  | | GTT | | GTG | | V | V | | | Synonymous | |  | | 3 | |  |
| 4462 | 55 | |  | | ATC | | ATT | | I | I | | | Synonymous | |  | | 3 | |  |
| 4489 | 1 | |  | | GAG | | GAT | | E | D | | | Non-Synonymous | | A-A | | 3 | |  |
| 4523 | 26 | |  | | CAG | | AAG | | Q | K | | | Non-Synonymous | | P-B | | 1 | |  |
| 4531 | 54 | |  | | GGC | | GGT | | G | G | | | Synonymous | |  | | 3 | |  |
| 4552 | 57 | |  | | CGC | | CGT | | R | R | | | Synonymous | |  | | 3 | |  |
| 4615 | 56 | | 1 | | CGC | | CGA | | R | R | | | Synonymous | |  | | 3 | |  |
|  |  | | 55 | | CGC | | CGT | | R | R | | | Synonymous | |  | | 3 | |  |
| 4623 | 1 | |  | | GAA | | GCA | | E | A | | | Non-Synonymous | | A-NP | | 2 | |  |
| 4630 | 6 | |  | | ACT | | ACC | | T | T | | | Synonymous | |  | | 3 | |  |
| 4633 | 3 | |  | | AAG | | AAT | | K | N | | | Non-Synonymous | | B-P | | 3 | |  |
| 4658 | 39 | | 7 | | TTA | | ATA | | L | I | | | Non-Synonymous | | NP-NP | | 1 | |  |
|  |  | | 32 | | TTA | | ATG(TTG) | | L | M | | | Non-Synonymous | | NP-NP | | 3 | |  |
| 4660 | 33 | | 32 | | TTA | | ATG(ATT) | | L | M | | | Non-Synonymous | | NP-NP | | 1 | |  |
|  |  | | 1 | | TTA | | TTG | | L | L | | | Synonymous | |  | | 3 | |  |
| 4667 | 33 | |  | | GAA | | TAA | | E | STOP | | |  | |  | | 1 | |  |
| 4679 | 2 | |  | | TAA | | CAA | | STOP | Q | | |  | |  | | 1 | |  |

| **CDS5** |  |  |  |  |  |  |  |  |  |
| --- | --- | --- | --- | --- | --- | --- | --- | --- | --- |
| Base | Number of SNPs | Number of SNPs in tri-allelic positions | P.Mutant code | SNP Change | Pre-AA | Post-AA | Synonymous/ Non-Synonymous SNP | Polar / Non Polar / Basic / Acidic | Codon Base Change |
| 4776 | 55 |  | GAA | CAA | E | Q | Non-Synonymous | A-P | 1 |
| 4811 | 28 |  | GGG | GGA | G | G | Synonymous |  | 3 |
| 4812 | 1 |  | CAA | AAA | Q | K | Non-Synonymous | P-B | 1 |
| 4858 | 135 |  | ACA | AAA | T | K | Non-Synonymous | P-B | 2 |
| 4871 | 1 |  | GTC | GTG | V | V | Synonymous |  | 3 |
| 4923 | 55 |  | TCA | CCA | S | P | Non-Synonymous | P-NP | 1 |
| 4977 | 1 |  | CTT | ATT | L | I | Non-Synonymous | NP-NP | 1 |
| 4998 | 165 |  | GAT | AAT | D | N | Non-Synonymous | A-P | 1 |
| 5011 | 55 |  | GAT | GGT | D | G | Non-Synonymous | A-P | 2 |
| 5038 | 58 |  | AGT | AAT | S | N | Non-Synonymous | P-P | 2 |
| 5055 | 1 |  | TTA | CTA | L | L | Synonymous |  | 1 |
| 5068 | 221 |  | AAA | ACA | K | T | Non-Synonymous | B-P | 2 |
| 5156 | 55 |  | AGT | AGG | S | R | Non-Synonymous | P-B | 3 |
| 5158 | 56 |  | AAC | AGC | N | S | Non-Synonymous | P-P | 2 |
| 5205 | 1 |  | ACA | TCA | T | S | Non-Synonymous | P-P | 1 |
| 5246 | 1 |  | GAT | GAC | D | D | Synonymous |  | 3 |
| 5270 | 1 |  | GGC | GGA | G | G | Synonymous |  | 3 |
| 5271 | 30 |  | GGC | AGC | G | S | Non-Synonymous | P-P | 1 |
| 5312 | 1 |  | CCC | CCA | P | P | Synonymous |  | 3 |
| 5314 | 53 |  | TGC | TAC | C | Y | Non-Synonymous | P-P | 2 |
| 5343 | 285 |  | ATT | GTT | I | V | Non-Synonymous | NP-NP | 1 |
| 5372 | 284 | 230 | AGT | AGC | S | S | Synonymous |  | 3 |
|  |  | 54 | AGT | AGA | S | R | Non-Synonymous | P-B | 3 |
| 5377 | 54 | 1 | ACT | AAT | T | N | Non-Synonymous | P-P | 2 |
|  |  | 53 | ACT | ATT | T | I | Non-Synonymous | P-NP | 2 |
| 5393 | 1 |  | ACT | ACA | T | T | Synonymous |  | 3 |
| 5396 | 195 |  | CCG | CCA | P | P | Synonymous |  | 3 |
| 5485 | 1 |  | ACA | ATA | T | I | Non-Synonymous | P-NP | 2 |
| 5510 | 285 |  | TTA | TTG | L | L | Synonymous |  | 3 |
| 5513 | 188 |  | GAG | GAA | E | E | Synonymous |  | 3 |

| **CDS6** |  |  |  |  |  |  |  |  |  |
| --- | --- | --- | --- | --- | --- | --- | --- | --- | --- |
| Base | Number of SNPs | Number of SNPs in tri-allelic positions | P.Mutant code | SNP Change | Pre-AA | Post-AA | Synonymous/ Non-Synonymous SNP | Polar / Non Polar / Basic / Acidic | Codon Base Change |
| 5621 | 53 |  | AGA | AGC | R | S | Non-Synonymous | B-P | 3 |
| 5625 | 1 |  | GTG | TTG | V | L | Non-Synonymous | NP-NP | 1 |
| 5714 | 2 |  | GTA | GTG | V | V | Synonymous | NP-NP | 3 |
| 5733 | 1 |  | TTA | CTA | L | L | Synonymous | NP-NP | 1 |
| 5860 | 1 |  | AAG | AGG | K | R | Non-Synonymous | B-B | 2 |
| 5870 | 27 |  | ATT | ATC | I | I | Synonymous |  | 3 |

| **CDS7** |  |  |  |  |  |  |  |  |  |
| --- | --- | --- | --- | --- | --- | --- | --- | --- | --- |
| Base | Number of SNPs | Number of SNPs in tri-allelic positions | P.Mutant code | SNP Change | Pre-AA | Post-AA | Synonymous/ Non-Synonymous SNP | Polar / Non Polar / Basic / Acidic | Codon Base Change |
| 5975 | 9 |  | GGA | GGG | G | G | Synonymous |  | 3 |
| 6065 | 36 | 20 | CCG | CCA | P | P | Synonymous |  | 3 |
|  |  | 16 | CCG | CCC | P | P | Synonymous |  | 3 |
| 6106 | 135 |  | AGT | AAT | S | N | Non-Synonymous | P-P | 2 |
| 6108 | 287 |  | GAC | AAC | D | N | Non-Synonymous | A-P | 1 |
| 6122 | 53 |  | TTG | TTA | L | L | Synonymous |  | 3 |
| 6125 | 1 |  | CAC | CAA | H | Q | Non-Synonymous | B-P | 3 |
| 6126 | 1 |  | GAC | AAC | D | N | Non-Synonymous | A-P | 1 |
| 6136 | 1 |  | TAC | TGC | Y | C | Non-Synonymous | P-P | 1 |
| 6139 | 136 |  | ACA | AAA | T | K | Non-Synonymous | P-B | 2 |
| 6145 | 1 |  | AAC | AGC | N | S | Non-Synonymous | P-P | 2 |
| 6211 | 286 |  | TCA | TTA | S | L | Non-Synonymous | P-NP | 2 |
| 6215 | 8 |  | TCC | TCT | S | S | Synonymous |  | 3 |
| 6233 | 1 |  | TTG | TTT | L | F | Non-Synonymous | NP-NP | 3 |
| 6249 | 10 |  | CCT | TCT | P | S | Non-Synonymous | NP-P | 1 |
| 6289 | 1 |  | GCT | GTT | A | V | Non-Synonymous | NP-NP | 2 |
| 6314 | 2 |  | ATA | ATC | I | I | Synonymous |  | 3 |
| 6379 | 58 |  | GCT | GTT | A | V | Non-Synonymous | NP-NP | 2 |
| 6550 | 9 |  | AAA | AGA | K | R | Non-Synonymous | B-B | 2 |
| 6593 | 35 |  | CTC | CTT | L | L | Synonymous |  | 3 |
| 6675 | 1 |  | CAT | TAT | H | Y | Non-Synonymous | B-P | 1 |
| 6731 | 1 |  | ACA | ACG | T | T | Synonymous |  | 3 |

| **CDS8** |  |  |  |  |  |  |  |  |  |
| --- | --- | --- | --- | --- | --- | --- | --- | --- | --- |
| Base | Number of SNPs | Number of SNPs in tri-allelic positions | P.Mutant code | SNP Change | Pre-AA | Post-AA | Synonymous/ Non-Synonymous SNP | Polar / Non Polar / Basic / Acidic | Codon Base Change |
| 6758 | 54 |  | GAT | AAT | D | N | Non-Synonymous | A-P | 1 |
| 6763 | 32 |  | GTC | GTT | V | V | Synonymous |  | 3 |
| 6769 | 2 |  | TTT | TTC | F | F | Synonymous |  | 3 |
| 6784 | 1 |  | ACT | ACA | T | T | Synonymous |  | 3 |
| 6794 | 55 |  | CTA | TTA | L | L | Synonymous |  | 1 |
| 6811 | 223 |  | ACG | ACA | T | T | Synonymous |  | 3 |
| 6817 | 282 | 281 | CCC | CCT | P | P | Synonymous |  | 3 |
|  |  | 1 | CCC | CCA | P | P | Synonymous |  | 3 |
| 6822 | 1 |  | ATT | ACT | I | T | Non-Synonymous | NP-P | 2 |
| 6857 | 3 |  | AGT | GGT | S | G | Non-Synonymous | P-P | 1 |
| 6873 | 54 |  | CGA | CAA | R | Q | Non-Synonymous | B-P | 2 |
| 6883 | 31 |  | TTA | TTG | L | L | Synonymous |  | 3 |
| 6904 | 1 |  | TCG | TCA | S | S | Synonymous |  | 3 |
| 6912 | 2 |  | GAA | GGA | E | G | Non-Synonymous | A-P | 2 |
| 6936 | 1 |  | GCG | GAG | A | E | Non-Synonymous | NP-A | 2 |
| 7038 | 2 |  | CAC | CTC | H | L | Non-Synonymous | B-NP | 2 |
| 7048 | 32 |  | TCA | TCG | S | S | Synonymous |  | 3 |
| 7087 | 1 |  | ACT | ACC | T | T | Synonymous |  | 3 |
| 7105 | 1 |  | AAT | AAC | N | N | Synonymous |  | 3 |
| 7117 | 2 |  | TAT | TAC | Y | Y | Synonymous |  | 3 |
| 7171 | 1 |  | TCG | TCA | S | S | Synonymous |  | 3 |
| 7201 | 7 |  | GCC | GCT | A | A | Synonymous |  | 3 |
| 7216 | 1 |  | GAT | GAC | D | D | Synonymous |  | 3 |
| 7237 | 1 |  | GTG | GTA | V | V | Synonymous |  | 3 |
| 7282 | 3 |  | GTG | GTT | V | V | Synonymous |  | 3 |
| 7317 | 1 |  | GAC | GGC | D | G | Non-Synonymous | A-P | 2 |
| 7343 | 2 |  | GAT | AAT | D | N | Non-Synonymous | A-P | 1 |
| 7347 | 134 |  | TCA | TTA | S | L | Non-Synonymous | P-NP | 2 |
| 7359 | 1 |  | CGC | CTC | R | L | Non-Synonymous | B-NP | 2 |
| 7399 | 195 |  | ATG | ATA | M | I | Non-Synonymous | NP-NP | 3 |
